# Spatial Inequity of Multi-Level Healthcare Services in a Rapid Expanding Immigrant City of China: A Case Study of Shenzhen

**DOI:** 10.3390/ijerph16183441

**Published:** 2019-09-17

**Authors:** Wei Hu, Lin Li, Mo Su

**Affiliations:** School of Resource and Environment Science, Wuhan University, 129 Luoyu Rd., Wuhan 430079, China; huwei_whu@whu.edu.cn (W.H.); mynameisyo@163.com (M.S.)

**Keywords:** healthcare services, inequity, accessibility, hierarchical medical system, Shenzhen city

## Abstract

Since the onset of reform and opening up in China, large cities in the nation have been experiencing problems related to limited medical resources. These resource limitations are due to rapid population growth and urban expansion. As the country’s fastest growing city, Shenzhen has experienced a substantial misalignment between the supply and the demand of healthcare services. Numerous researchers have analyzed spatial inequity in healthcare services by focusing on the spatial accessibility of medical facilities, such as hospitals, clinics, and community health service centers (CHSCs). However, the issue of inequity in healthcare services for vulnerable groups has largely been ignored. We chose general hospitals (GHs) and CHSCs, which provide direct healthcare services to residents, as the study objects. By performing spatial accessibility analysis using the gravity model and the two-step floating catchment area method, we investigated healthcare services inequity for vulnerable groups based on four dimensions: residential type, age, education level, and occupation. We found that the services provided by GHs cannot meet the demand in Shenzhen. This inadequacy is characterized by spatial centralization and neglect of those who reside in urban villages, who have low education levels, and who are employed in the manufacturing industry. In contrast, CHSCs generally serve a relatively broad population. This phenomenon is related to differences in the land and capital needs between GHs and CHSCs. Our study reveals that an appropriate adjustment of GH location could significantly improve healthcare services inequity. Therefore, to alleviate this inequity, it is particularly necessary to increase the number of GHs in the peripheral circle and in areas with large vulnerable populations, accelerate the implementation of the hierarchical medical system, and promote the transfer of medical resources to grassroot institutes through CHSCs. This study helps improve our understanding of healthcare services inequity in rapid expanding cities, which is of substantial significance for improving the planning and construction of medical facilities, facilitating scientific decision-making, and promoting social equity.

## 1. Introduction

Medical facilities are vital for the health of all residents. Thus, priority must be awarded to facilities that provide healthcare services. However, with the rapid urbanisation and the influx of migrants, problems of insufficient supply and inequity in healthcare services have become common in China’s megacities. Studies have shown that an adequate supply of medical resources can reduce the risk of diseases, such as cardiovascular disease [1]. Inequity in healthcare services increases the differences in health status and quality of life among urban residents [2]. Therefore, the Chinese government has attached substantial importance to the construction and development of medical facilities. China’s National Planning Guideline for the Health Service System (2015–2020) proposed prioritising the accessibility of basic medical facilities while promoting equity, establishing and perfecting a hierarchical medical system, and coordinating the allocation of medical resources of all types and levels in different regions.

The most direct way to evaluate inequity in healthcare services is to consider the residents in a region as an entire population and use the corresponding indicators to measure the differences in the healthcare service levels of the population. Generally, researchers tend to directly analyse the differences in service levels between regions [3,4] or calculate the standard deviation of the accessibility of medical facilities, whereby the larger the standard deviation is, the stronger the inequity is [5]. Researchers have also used the Gini coefficient or the Theil index to analyse the inequity in healthcare services between different regions [6,7]. However, in reality, inequity in healthcare services is often closely related to various attributes of the population, such as income, age, education level, and race. One goal of the allocation of medical facilities should be to minimise service-level differences between different population groups. In western countries, such as the United States, for historical and cultural reasons, studies of equity often consider dimensions such as race, ethnicity, and economic and social factors (i.e., income and gender) [8,9]. In China, particularly in large cities with histories of rapid urbanisation, differences within the population are mainly reflected in social and economic factors, including household registration, income, education level, and occupation [10].

As a typical representative of cities that have undergone rapid urbanisation since the onset of reform and opening up, Shenzhen has exhibited rapid urban expansion and population growth since the 1980s. From 1980 to 2016, the city’s resident population has increased by 35.77 times, with an average annual increase of 10.45%. This population growth is on-going. Compared with the population growth, the construction of medical facilities in Shenzhen clearly lags behind. Shenzhen’s hospital beds per capita are lower than the national average, and the city is suffering from a conspicuous insufficiency of medical resources. After many years of continuous population growth, the non-resident population in Shenzhen accounts for 69% of the total population. This non-resident population is generally characterised by low education levels, low incomes, low residential stability, and young age [11,12]. Because of the high property price, most such non-residents face difficulties in securing housing through the formal housing market. Consequently, urban villages have become areas with concentrated migrant populations, a phenomenon caused by certain advantages that the villages offer, such as low house rent and convenient locations. Gradually, a spatial pattern in the city has emerged that reflects the difference between resident and non-resident status [13,14]. The non-resident populations are relatively vulnerable groups, have a low ability to withstand risk, and demand special attention with respect to healthcare services.

The goal always pursued by the Chinese government is equalisation. In the case of unified medical policies and cost standards in the whole city, spatial accessibility is the key factor that restricts the healthcare service level. However, few studies have discussed the equity of vulnerable groups from the perspective of accessibility of healthcare services. The goal of this study is to analyse the equity of healthcare services among different population groups and grasp the equity problem caused by the layout of medical facilities, which can provide a basis for research on medical facility layout optimisation and social equity and can provide a decision-making basis for the government to optimize the layout of medical facilities. Because of the strong correlation between residential conditions and income, four dimensions (residential type, age, education level, and occupation) were selected in this study to assess the accessibility of residents to medical facilities in different groups and to analyse the spatial distribution pattern of medical facility accessibility. Based on this, we discuss the inequity in healthcare services and make recommendations for improvements.

## 2. Materials and Method

### 2.1. Study Area

**Figure 1:**
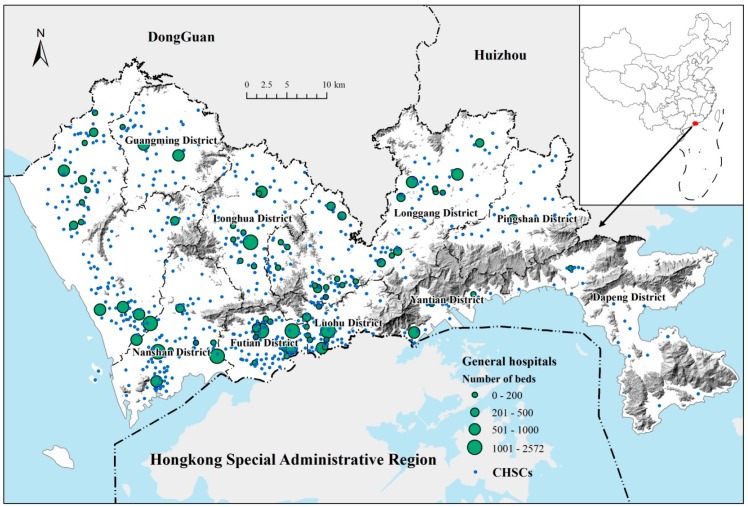
The overview of Shenzhen city.

Shenzhen is located in the south of China, bordering Dongguan and Huizhou to the north, Hong Kong to the South, and Zhujiang Estuary to the West, and is a mega-city in Southern China with a land area of 1997 km^2^ and a resident population of 11.91 million (end of 2016) [15]. It is a special economic zone established in 1980 along with China’s reform and opening up policy [16]. The city comprises 10 districts. The Futian, Luohu, Nanshan, and Yantian districts adjacent to Hong Kong in the south are the earliest special zones. They are also the first areas where construction and development took place, and the public facilities are the most mature. The city has formed a two-level central structure. The main centre is the Futian-Luohu centre, and the sub-centres mainly include the Nanshan centre, the Baoan centre, and the Longgang centre. In the construction of medical facilities, Shenzhen has established a “GH-CHSC” two-level healthcare service system. CHSCs serve community residents, are equipped with a small number of general practitioners, provide primary health care services, are generally responsible for the treatment and prevention of common diseases, and are located in the community. GHs are large-scale hospitals with various professional departments, providing secondary and tertiary medical care and hospitalisation services. All the CHSCs and most of the GHs are established and maintained by the government, and both of them use the same government guidelines in health insurance policy and medical expenses. As of 2016, there are 81 GHs and 591 CHSCs in the city (Figure 1).

### 2.2. Data Collection

The data used in the study mainly include medical facilities, population, roads, buildings, and spatial units. All the data were collected from official sources. (1) Medical facilities include GHs and CHSCs. The data came from the Shenzhen Municipal Health Commission. The GH data included spatial coordinates, names, and bed numbers. The CHSC data included spatial coordinates, names, and doctor numbers. (2) The population data were obtained from the Shenzhen Social Work Committee. The data were collected by about 15,000 community investigators throughout the city. They marked the population of the city according to different genders, ages, education levels, occupations, and other dimensions and recorded the population of each attribute combination in each spatial unit (i.e., “gender: male, age: 20–29, education: bachelor degree, occupation: manufacturing, number: 30”). (3) The road data were obtained from the Shenzhen Planning and Natural Resources Bureau, which was obtained from the first national survey of national geographic conditions in Shenzhen. The data contained vector data of all roads in the city, including road names and road grades. (4) The building data came from the Shenzhen Planning and Natural Resources Bureau, which was obtained from the annual building survey, including the spatial location, function, and building area of each building in the city. (5) Spatial unit data were obtained from Shenzhen Spatial Geographic Information Center. These data included basic units of grassroots management, including 9319 units, with an average area of 0.21 km^2^. They were used as basic units of accessibility analysis.

### 2.3. Data Pre-Treatment

Each type of data was processed to meet our research needs as follows.

(1) Spatialization of medical facility data. The table data of the GHs and CHSCs were converted to GIS point data based on spatial coordinates, and the spatial locations were verified based on the address information.
(2) Decomposition of residential types of the population. According to the classification provided in the “Shenzhen Urban Planning Standards and Guidelines” (2014), the residential buildings in the city were divided into four categories: R1, R2, R3, and R4. R1 included low-rise residential buildings and townhouses, typically with high housing value. R2 included multi-story and high-rise residential buildings. Such structures are commonly occurring commercial residential buildings. R3 included company-provided sleeping facilities or dormitories and apartments for singles. R4 included self-constructed residential houses in urban villages, typically with relatively poor living environments. As per the survey results of Shenzhen Urban Planning, the standard per-capita living areas of R1, R2, R3, and R4 type residential buildings are approximately 60, 31.25, 20, and 20 m^2^, respectively. Therefore, we can use the building data of the city to decompose the residential population. For each spatial basic grid, the decomposition method is as follows:
  $$P_{Ri}=P\cdot \frac{P_{Ri}^{\prime }}{P^{\prime }}$$
  where *P_Ri_* is the number of residents in each type of building *Ri* (*i* = 1, 2, 3, 4) within the grid, *P* is the total number of residents in the grid, $P_{Ri}^{\prime }$ is the number of residents that can be accommodated in each type of building *Ri* (*i* = 1, 2, 3, 4) in the grid, calculated based on the standard per-capita building area, and *P*′ is the total number of residents that can be accommodated, calculated based on the standard per-capita building area for all residential buildings in the grid.
(3) Dimensions of population data in a spatial unit. The populations within each spatial unit were divided based on the four dimensions of residence type, age, education level, and occupation, and the number of residents of each dimension in the spatial unit was calculated (Table 1).

## 3. Methods

The definition of the accessibility of facilities can be of various types, such as supply–demand ratio, distance to the nearest supply point, and average distance to a series of supply points [17], where the supply–demand ratio is the most commonly used definition and is usually calculated by the two-step floating catchment area method (2SFCA). In recent years, many scholars have proposed a series of improvements while using the method. For example, the enhanced two-step floating catchment area method was proposed to consider the service attenuation within the service area [18,19]. The dynamic service area method was proposed to consider different service radii [20]. The multi-traffic mode was introduced to distinguish different groups of people that have different travel characteristics [21].

We adopted the supply–demand ratio as an indicator to measure the accessibility of medical facilities and applied different measurement models for GHs and CHSCs. GHs are open to all residents. Patients typically visit GHs by a private vehicle, taxi, or public transportation. Residents who live far away from a GH are less likely to visit a GH. We applied the gravity model to evaluate GH accessibility and used the traffic network model to calculate the travel time. This method was originally developed and optimised by Weibull [22] and has gradually become widely used to analyse medical accessibility [23], commuting patterns [24], park accessibility [25], and evacuation sites [26]. The calculation method is as follows:

(1) $$A_{i}=\overset{n}{\underset{j=1}{\sum }}\frac{S_{j}}{d_{ij}{}^{\beta }V_{j}}V_{j}=\overset{m}{\underset{k=1}{\sum }}\frac{D_{k}}{d_{kj}{}^{\beta }}$$

where *A_i_* represents the spatial accessibility of demand point *i* to all supply points, *S_j_* represents the service capacity of supply point *j*, *D_k_* represents the demand scale of demand point *k*, *V_j_* represents the demand scale influencing factor, *d_ij_* represents the travel impedance (distance or time) between demand point *i* and supply point *j*, *β* represents the coefficient of friction of travel (which is set as 1.5 in this study), and *n* and *m* represent the number of supply points and demand points, respectively.

CHSCs are open to neighbourhood residents, with a service radius generally within 500 m. Residents mostly visit CHSCs on foot. Because of the small scope of services such facilities offer, there was no need to consider service degradation. We adopted a two-step floating catchment area method to calculate CHSC accessibility. Because of the sparse population distribution in some areas, we used 800 m as the search range. This method was first developed by W. Luo et al. by modifying FCA [27] and has been applied extensively in various countries and regions [28,29]. The equation is as follows:

(2) $$A_{i}=\underset{j\in \left\{d_{ij}\leq d_{0}\right\}}{\sum }R_{j}=\underset{j\in \left\{d_{ij}\leq d_{0}\right\}}{\sum }\frac{S_{j}}{\sum_{k\in \left\{d_{kj}\leq d_{0}\right\}}D_{k}}$$

where *A_i_* represents the supply–demand ratio of demand point *i*, *d_ij_* represents the distance between demand point *i* and supply point *j*, *R_j_* is the ratio of the facility size of supply point *j* to the population served within search radius *d*_0_, *S_j_* represents the supply scale of supply point *j*, and *D_k_* represents the demand scale of demand point *k*.

At present, the number of beds per 1000 individuals is used as the basis for assessing GH service capacity in China. Because CHSCs only provide outpatient services, the number of physicians per 10,000 individuals is used to evaluate CHSC service capacity. According to China’s National Planning Guideline for the Healthcare Service System (2015–2020), the number of physicians per 10,000 individuals in grassroots institutes (such as CHSCs) must be 2 by 2020. Shenzhen City Planning Standards and Guidelines (2014) specify a goal of 5 beds per 1000 individuals in GHs. Therefore, we used beds per 1000 individuals and physicians per 10,000 individuals to evaluate the spatial accessibility (supply–demand ratio) of GHs and CHSCs, respectively.

First, we used the spatial units as the basic units to calculate the accessibility distribution of GHs and CHSCs. Then, we used the population-weighted method to analyse the distribution, with sub-districts and administrative districts as basic units, to understand the overall accessibility of medical facilities in the entire city. Thus, we compared the accessibility of medical facilities of different populations with respect to various dimensions to discuss inequity issues.

## 4. Results

### 4.1. Spatial Accessibility of Medical Facilities

In 2016, there were 81 GHs in Shenzhen, including 30,702 beds. The average number of beds in each GH is 379.0, and the beds are mainly distributed in Longgang, Baoan, Futian, and Luohu districts. At the hospital scale, the hospitals in the Futian and Luohu districts of the municipal centre were the largest, with an average of 601.4 beds and 474.1 beds per hospital, respectively. Larger hospitals often indicate better medical service standards in China, so GHs had a spatial agglomeration tendency in the city centre. There were 591 CHSCs in the city, with a total of 3283 doctors. Each CHSC has 5.6 doctors on an average.

**Figure 2:**
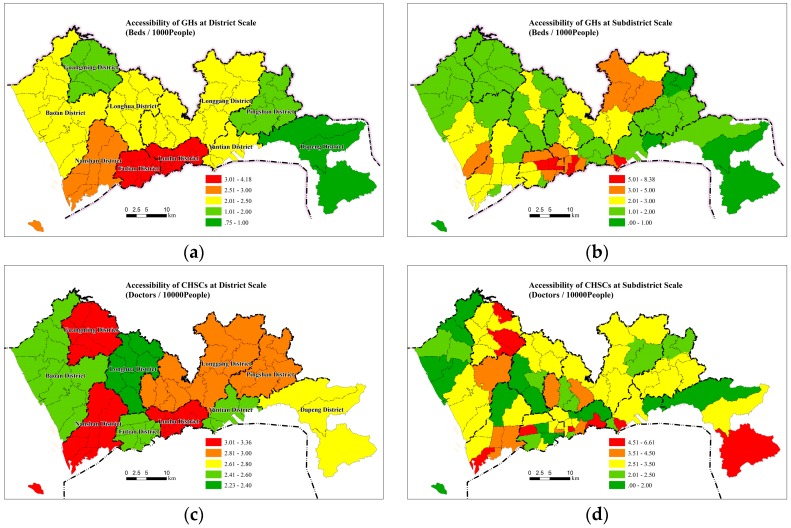
Spatial accessibility of GHs and CHSCs. (**a**) Spatial accessibility of GHs at district scale; (**b**) Spatial accessibility of GHs at subdistrict scale; (**c**) Spatial accessibility of CHSCs at district scale; (**d**) Spatial accessibility of CHSCs at subdistrict scale.

The gravity model and 2SFCA model were used to calculate the supply–demand ratios of GHs and CHSCs, respectively. The models were implemented by ArcGIS software. The GH supply–demand ratio in Shenzhen is 2.58 beds per 1000 individuals, which is only 51.6% of the five beds per 1000 target specified in the Shenzhen City Planning Standards and Guidelines (2014). The proportion of the population with a supply–demand ratio of more than five beds per 1000 individuals is only 8.08%, and the proportion of the population with a supply–demand ratio of less than 2.5 beds per 1000 individuals is 66.37%. These findings indicate that the overall service capacity of GHs in Shenzhen is seriously inadequate. The GH supply–demand ratio exhibits significant spatial differentiation (Figure 2). More specifically, there are significant differences between the inner and outer regions of the original special economic zones (SEZs) and between the central and western regions of the city. The GH supply–demand ratio is basically consistent with the level of economic development. On the administrative district scale, the supply–demand ratios of Futian, Luohu, and Nanshan in the original SEZ are the highest, all exceeding 2.5 beds per 1000 individuals. These three districts are also the areas with the highest per-capita GDP in Shenzhen. The GH supply–demand ratios in Dapeng and Pingshan in the east and Guangming in the northwest are less than one bed per 1000 individuals. These three districts have scarce medical resources, and the residents in these areas must commute longer to access healthcare services. On the sub-district scale, high GH supply–demand ratios are concentrated in a few small areas, namely, the neighbourhoods surrounding the city centre and the district centres of Luohu, Yantian, Bao’an, and Longgang. This pattern displays obvious characteristics of administrative guidance.

The supply–demand ratio of CHSCs in Shenzhen is 2.76 physicians per 10,000 individuals, which is higher than the target specified in the national planning documents. The proportion of the population with a supply–demand ratio above two and four physicians per 10,000 individuals is 58.88% and 14.12%, respectively. The accessibility of CHSCs is much better than that of GHs. However, the accessibility of nearly half of the population is still lower than the national standard, exhibiting room for improvement. The spatial distribution (Figure 2) of the CHSC supply–demand ratio does not display an obvious pattern on the administrative district scale or the sub-district scale. The Guangming, Dapeng, and Pingshan districts have relatively poor accessibility to GHs but fairly good accessibility to CHSCs.

### 4.2. The Relationship between Medical Facility Accessibility and Location

**Figure 3:**
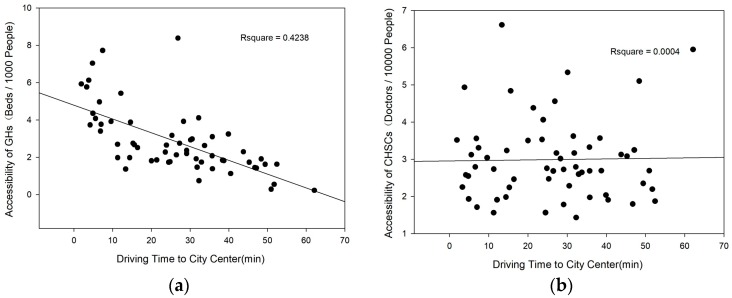
The relationship between driving time to city center and the accessibility. **(a)** The relationship between driving time to city center and the accessibility of GHs; (**b**) The relationship between driving time to city center and the accessibility of CHSCs.

We applied the traffic network model, using the sub-district as the basic unit, to calculate the driving time from the centre of each individual sub-district to the nearest municipal-level commercial service centre. The SPSS software (IBM, New York, NY, USA) and linear regression model were used to explore the relationship between medical facility accessibility and the driving time to the city centre. The R-squares for the GH and CHSC models are 0.4238 and 0.0004, respectively. Regression analysis (Figure 3) shows that the GH supply–demand ratio is strongly negatively related to the driving time to the city centre. Overall, the GH supply-to-demand ratio decreases by 0.74 beds per 1000 individuals for every 10-min increase in the driving time to a municipal-level commercial service centre. In contrast, the CHSC supply–demand ratio is not correlated with the driving time to a municipal-level commercial service centre. Therefore, because of the characteristics of GHs, GH resource allocation in Shenzhen remains substantially affected by spatial location, while CHSC resource allocation is not. A GH occupies a large area of land, requires a large one-time investment, and requires a long time from planning to site selection to construction and actual operation. Therefore, it is difficult for a new GH to provide services in a short time. In Shenzhen, the commercial service centres are located in areas that were developed earlier, with earlier construction and the highest degree of social development. Therefore, these areas present relatively good conditions for GH construction. For example, the Shenzhen People’s Hospital was constructed in 1946. Since the establishment of the Shenzhen Special Economic Zone in 1979, this hospital has undergone several expansions and gradually grown to its current service scale. In 2010, the SEZs in Shenzhen were extended to the entire city, and the investments on medical facilities in districts outside the original SEZs gradually increased. However, GHs still require a long time to reach the same service level as the hospitals within the original SEZs. In addition, the shortage of land resources in Shenzhen in recent years has added obstacles to GH construction. In contrast, CHSCs generally do not require a separate property and can be established through residential reconstruction and the renovation of existing housing. CHSCs are relatively small and can easily offer service capacity in a relatively short time. Therefore, CHSCs can be constructed in all districts in Shenzhen.

### 4.3. Spatial Accessibility of Different Type of Residents

Based on the assessment results for the accessibility of the spatial grids, we analysed the accessibility of medical facilities in four dimensions: residence type, age, education level, and occupation.

**Figure 4:**
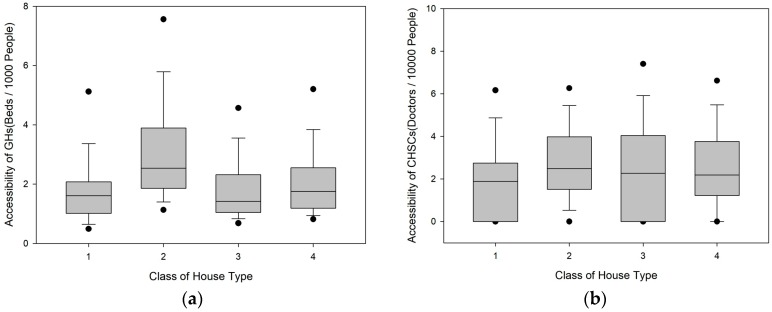
Box-plots of the accessibility for different classes of living house type. (**a**) Box-plots of the accessibility of GHs; (**b**) Box-plots of the accessibility of CHSCs.

As shown in Figure 4, the GH supply–demand ratio of R2 type residents is significantly higher than that of the other three categories and is 31.79% higher than the city average. R1 type residents have the lowest GH supply–demand ratio. For CHSCs, there is no significant difference in the supply–demand ratio among the R2, R3, and R4 type residents. However, the ratio of R1 type residents is significantly lower than the city average. The GH and CHSC supply–demand ratios of R1 type residents are 26.86% and 29.43% lower than the city average, respectively. This outcome is related to the fact that R1 residential buildings are generally located in neighbourhoods with substantial green areas and are thus separated from other types of housing.

**Figure 5:**
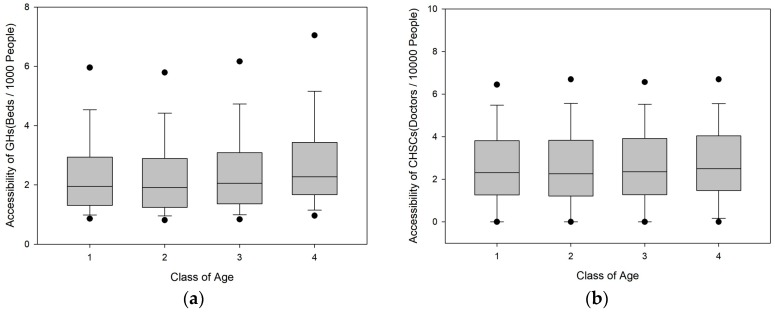
Box-plots of the accessibility for different classes of age. (**a**) Box-plots of the accessibility of GHs; (**b**) Box-plots of the accessibility of CHSCs.

As shown in Figure 5, as the age of residents increases, the GH supply–demand ratio exhibits an upward trend to a certain extent, while the CHSC supply–demand ratio does not display a significant difference between different age groups. It is worth noting that the GH and CHSC supply–demand ratios are both the highest for populations over 60 years old: 18.44% and 6.40% higher than the city average, respectively. This phenomenon may be related to two factors. First, because of the relatively high housing prices, young immigrants typically live in areas near the city centre, and populations with relatively older ages are more concentrated in city centre areas with good GH accessibility. Second, the elderly population requires more healthcare services and tends to live in areas more accessible to medical facilities.

**Figure 6:**
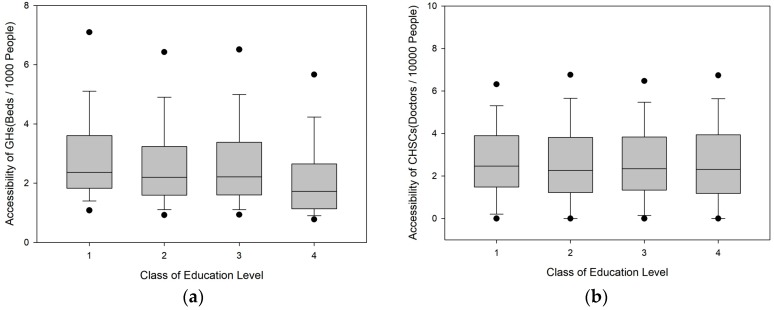
Box-plots of the accessibility for different classes of education level. (**a**) Box-plots of the accessibility of GHs; (**b**) Box-plots of the accessibility of CHSCs.

As shown in Figure 6, the GH supply–demand ratio displays a significant downward trend with a decrease in education level. The GH supply–demand ratios of individuals with bachelor’s degrees or higher, junior-college degrees, and high-school education are 22.46%, 12.61%, and 12.01% higher than the city average, respectively, while that of the population with a junior-high school education and below is 10.18% lower than the city average. The CHSC supply–demand ratio does not exhibit a significant correlation with education level. The population with bachelor’s degrees or higher has the highest ratio, which is only 3.10% higher than the city average.

**Figure 7:**
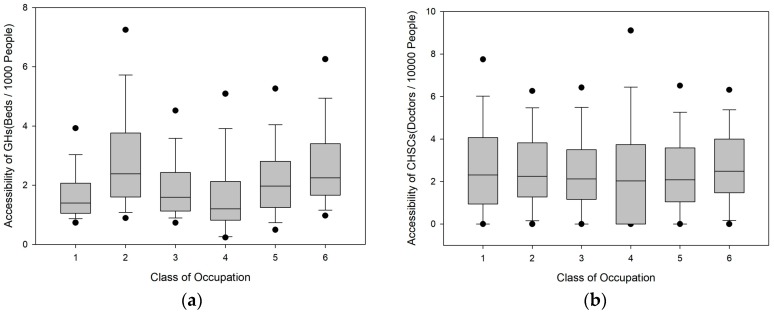
Box-plots of the accessibility for different classes of occupation. (**a**) Box-plots of the accessibility of GHs; (**b**) Box-plots of the accessibility of CHSCs.

As shown in Figure 7, the GH supply–demand ratio varies greatly among populations with different occupations. Specifically, the populations employed in manufacturing, transportation, agriculture, and construction have supply–demand ratios that are 29.70%, 18.92%, 33.97%, and 9.86% lower than the city average, respectively, whereas the ratios of the population employed in commercial services and the unemployed population are 24.20% and 12.34% higher than the city average, respectively. This phenomenon is related to the distribution pattern of different occupations in the city. Commercial service enterprises are generally distributed in various city centre areas, and the residential areas of the unemployed population are also close to the city centre. These areas have relatively high GH service capacities. Transportation, agriculture, and construction enterprises are often distributed in the peripheral areas of the city. According to demographic characteristics, Shenzhen’s unemployed population often includes full-time wives or elderly persons living with their children, and their unemployment status does not affect their quality of life. The CHSC supply–demand ratio does not vary much among populations with different occupations. Only the populations employed in the construction and transportation industries display relatively large deviations. The CHSC supply–demand ratios for these two populations are 8.01% and 6.43% lower than the city average, respectively.

Calculating the proportion of the population in each dimension in the spatial grid can reveal the demographic characteristics within each grid. Subsequent multi-variant regression analysis can reveal the relationships between these characteristics and accessibility to medical facilities. To enhance the explanatory significance of the analysis result, in the regression, we excluded population types that constitute a small proportion of the population (such as R1 type residents), or we merged populations that constitute small proportions of the population in the regression. Ultimately, we have five variables: the proportion of R4 type residents (PR4), the proportion of the population below 40 years old (PY40), the proportion of the population with a college degree or higher, the proportion of the population employed in the manufacturing industry (PM), and the proportion of the population employed in commercial services. A total of 7127 grids with more than 100 individuals were selected as the regression samples. The GH and CHSC supply–demand ratios were subjected to regression analysis with the selected variables. The SPSS software and multiple linear regression model were used to carry out the analysis. The R-squares for the GH and CHSC models are 0.1230 and 0.0050, respectively. The results are shown in Table 2.

The GH supply–demand ratio is significantly negatively correlated with PR4, PY40, and PM and significantly positively correlated with PJ and PB. These outcomes indicate that R4 type residents, young individuals, and individuals employed in the manufacturing industry are at a disadvantage in accessing healthcare services at GHs, while individuals with high education levels and those employed in the commercial service industry enjoy advantages in obtaining such services. The CHSC model is not as significant as GHs; the supply–demand ratio of CHSC is negatively correlated with PY40 and positively related with the other four population types. These outcomes indicate that R4 type residents, older individuals, individuals with high education levels, and individuals employed in manufacturing and commercial services have more access to healthcare services at CHSCs. R4 type residents are mostly low-income individuals and migrants employed in the manufacturing industry. These individuals represent a vulnerable population and to a certain extent have acquired health protection from CHSC services. Future allocation of GH resources should also consider serving these vulnerable groups.

### 4.4. Sensitivity of Inequity of Healthcare Services to Hospital Layout

**Figure 8:**
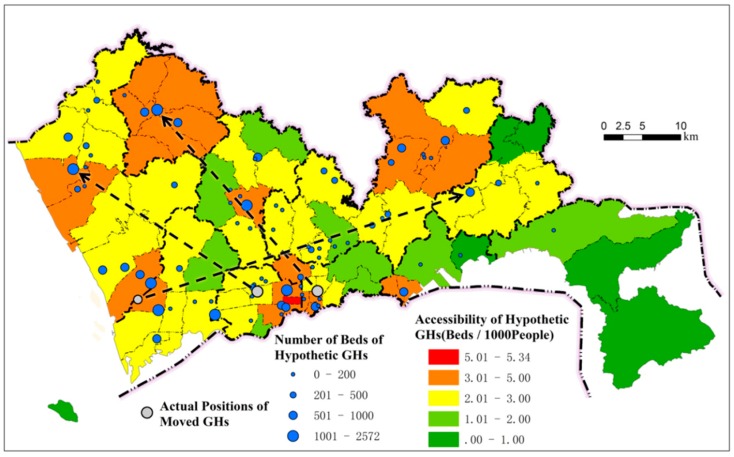
Spatial accessibility of hypothetic GHs.

To further investigate the relationship between hospital location distribution and the equity of healthcare services, we selected three large hospitals in areas with relatively high accessibility and assumed that they were located in peripheral areas with a relatively low GH supply–demand ratios to evaluate the distribution of accessibility. The three hospitals are located in Luohu District, Futian District, and Nanshan District, and the numbers of beds are 2572, 1600, and 1000, respectively, accounting for 16.8% of the total number of GH beds in Shenzhen. The hypothetic distribution is shown in Figure 8. By comparing with Figure 2, we can note that the overall distribution of the GH supply–demand ratio is more balanced, and the services in the peripheral areas are significantly improved. The Gini coefficient of the GH supply–demand ratio in the entire city decreases from 0.356 in reality to 0.225 under the hypothetic distribution conditions, indicating that the hypothetic distribution can substantially increase the overall equity of healthcare services.

Under the hypothetic distribution, the equity of GH services significantly increases for all population types. The difference between the highest and lowest accessibility for the four dimensions—residential type, age, education level, and occupation—decreased by 52.7%, 80.2%, 84.3%, and 42.5%, respectively, and the difference was not significant for populations with different ages and education levels (Table 3). Therefore, a spatial adjustment of only 16.8% in GH services in Shenzhen could substantially improve equity in healthcare services.

## 5. Discussion

### 5.1. Spatial Inequity of Healthcare Services

The accessibility assessment in this study revealed that the supply–demand ratio of GHs and CHSCs for different regions and population types varies significantly. The distribution of the supply–demand ratios of GHs and CHSCs exhibits completely different characteristics spatially and among different population groups. GHs are more accessible for R2 type residents, those of older age, those with high education levels, and individuals employed in commercial services. They are less accessible for R1 and R4 type residents, those with junior-high school education and less, and those employed in the manufacturing and agricultural industries. CHSCs are more accessible for R2 and R4 type residents and those over 60 years of age. They are less accessible for R1 type residents. Generally, GH accessibility exhibits population selectivity for the four dimensions investigated in this study, whereas CHSC accessibility displays weak population selectivity.

According to the Shenzhen Statistical Yearbook 2017, the average wages of the construction industry, agricultural and breeding industry, manufacturing industry, transportation industry, and service and business industry in 2016 were 64.1k, 76.5k, 77.4k, 97.3k, and 111.3k Yuan, respectively. The wages of the construction industry, agricultural and breeding industry, and manufacturing industry were lower than the average of 89.8k Yuan in the whole city [15]. We defined the people who are engaged in the construction industry, agricultural and breeding industry, manufacturing industry, who live in urban villages (R4 type), and who have a lower educational background as relatively vulnerable groups. The vulnerable groups experience greater inequity in GH services and enjoy relatively fair treatment in the service of CHSCs, which means that it is easier for them to receive primary medical services, but more difficult to receive secondary and tertiary medical services.

### 5.2. Planning and Construction Proposals for Medical Facilities

The medical facility accessibility distribution is caused by the interaction of urban planning, urban construction, and population migration. China’s land use planning highly emphasises the overall average level over equal distribution of public resources [30,31]. The planners generally plan facilities from the perspective of increasing the total facility supply and improving the overall equity of healthcare services. However, in the actual process of construction and development, the funds and land used for facility construction are restricted by the financial resources of the municipal and district governments, which causes differences in the scale of the facilities and the construction time in different regions within a certain period of time, gradually resulting in spatial inequity. Because of the differences in factors such as income, age, education level, and occupation, urban residents have vastly different needs in selecting residential locations and healthcare services. Residents with better economic conditions and higher medical needs (i.e., older people) tend to live in areas with better access to medical facilities. These different needs can further increase healthcare service inequity.

Based on the preceding analysis, the inequity of GH medical services is highly sensitive to the spatial distribution of GHs. Construction of new large GHs in peripheral areas could effectively reduce the inequity of healthcare services for various population types. Therefore, our study offers the following suggestions for the planning and construction of medical facilities. First, GH planning and construction should be substantially strengthened. In particular, GHs should be planned and constructed in densely populated areas outside the original SEZs, near urban villages, in areas with dense populations of residents with low education levels, and areas with numerous manufacturing enterprises. Second, the number of CHSCs should continue to increase in the areas that lack CHSC services. Third, the CHSC service capacity should be improved and the hierarchical medical system should be further promoted. Because GH service capacity cannot be rapidly increased in a short time, high-quality healthcare service resources should be transferred from GHs to CHSCs to provide convenient healthcare services to residents by taking advantage of the better spatial distribution of CHSCs.

### 5.3. Advantages and Limitations of the Study

In terms of the methods, the gravity model and 2SFCA method are widely used, and the 2SFCA method can be regarded as a special case of the gravity model with limited service scope and without considering distance decay. For GHs, according to the National Medical and Health Service System Planning Outline (2015–2020), the service radius of GHs is about 50 km, whereas the longest span of densely populated areas in Shenzhen is within 60 km, so it is not necessary to set the service radius for GHs. At present, the E2SFCA method is used to consider distance decay in the service radius [32,33]. If the service radius was enlarged to the whole study area, it would become a gravity model. Therefore, the use of the gravity model directly in this study was appropriate. The service radius of CHSCs is basically less than 800 m. Residents can walk to a CHSC and ignore the distance decay effect. Therefore, the method system selected in this study is suitable for evaluating the two-level healthcare service system in Chinese cities.

Previous studies on the accessibility of medical facilities in Shenzhen have confirmed the spatial agglomeration effect of the accessibility of GHs [34,35], which is the same as the result of this study. In fact, the service agglomeration of large hospitals is prevalent in many cities [36,37]. However, these studies regard the population as a homogeneous whole, so it is impossible to observe the inequity of healthcare services among different types of populations [38]. Compared with previous studies, this study has the following advantages. First, the population is decomposed into four dimensions, so we can observe the accessibility of medical facilities of different population groups. Second, the GHs and CHSCs, which constitute the hierarchical medical system in Chinese cities, are both considered, and significant differences in service equity are found between them, which provides a decision-making basis for the improvement of medical systems. Third, the sensitivity of inequity of healthcare services to hospital layouts has been proven, so an increase in the number of GHs in some areas can greatly alleviate the problem of equity. This provides an important reference for the planning and site selection of new GHs.

However, there are several limitations to this study, which can be further improved in the future. First, we calculated travel time using a road traffic network model, without considering the choice of multiple modes of transportation, which can be improved by introducing the commuting time in multiple travel modes provided by internet map services [34]. Second, in some cases, patients will choose a hospital that is farther away but larger in size than the nearest one, which is different from the result of classic models. To solve this problem, it is necessary to introduce patient visiting data to modify the classic models.

## 6. Conclusions

Selecting GHs and CHSCs that provide direct healthcare services to residents as our study subject, we applied the gravity model and the two-step floating catchment area method to evaluate the spatial accessibility of medical facilities (supply–demand ratio). Based on this, we divided the population of the entire city into different population types as per four dimensions—residence type, age, educational level, and occupation—to investigate inequity in healthcare services among different population groups under the current medical facility and population distribution conditions. In addition, we elucidated the reasons for the inequity. We found that the service capacity of GHs in Shenzhen does not meet the demand. Medical resources are concentrated in the city centre, and vulnerable groups, such as the residents of urban villages, those with low education levels, and individuals employed in the manufacturing industry, have low access to healthcare services. At present, the hierarchical medical system has not been effectively implemented, and GHs remain the main source of healthcare services for residents. Under the current circumstances, the previously mentioned vulnerable groups must pay higher commuting costs to access medical resources, which is highly unfavourable to resident health and social equity. In contrast, the CHSC situation can be viewed with relative optimism. Except for a lack of services for R1 type residents, CHSCs did not exhibit obvious inequity issues for providing healthcare services to all population types. In fact, CHSCs actually provide higher accessibility to some vulnerable groups, including individuals residing in urban villages and individuals employed in manufacturing. The main challenge for CHSCs is to further strengthen their service scope. Based on these conclusions and the inherent characteristics of GH construction and development, we recommend that urban planners strengthen GH availability in areas outside the city centre and in areas with concentrations of vulnerable groups, increase the number of CHSCs in areas with insufficient healthcare services, and accelerate the implementation of the hierarchical medical system.

Since the onset of reform and opening up, Shenzhen has been the fastest-growing city in China and has become an international megalopolis that attracts a large number of migrants from across the country every year [39]. The successful economic development of Shenzhen has become a model for many developing countries. However, it is worth noting that with the rapid urban expansion and population growth, Shenzhen’s city centre has attracted high-quality medical resources because of its advantages with respect to location and funding availability. Thus, a central city-peripheral area duality has gradually emerged. This duality has a strong coupling relationship with residential type, age, education level, and occupation, resulting in inequity in healthcare services among the different population groups. At present, Shenzhen’s government plans to add 25,000 new beds between 2016 and 2020, and Shenzhen Medical Institutions Planning 2016–2020 advises planning based on fairness and scientific allocation. However, because of the rapid expansion and lack of land resources, it is difficult to find appropriate construction sites for large new hospitals. Therefore, reasonable advance planning of large medical facilities is particularly important. Our analysis of equity in access to medical facilities in Shenzhen will serve as an important reference in the future planning and construction of medical facilities and provide lessons and experiences for rapidly growing cities in China and other developing countries. The research framework and methodology not only are applicable to most Chinese cities but also provide an important reference for research in other countries and regions. In the future, this study can be further improved by introducing the patient visiting data and internet map services.

## Figures and Tables

**Table 1:** The classification of residents and the proportion of different types.

Dimensions	Type	Class	Proportion of the Population
House type	R1	1	0.31%
	R2	2	32.51%
	R3	3	15.10%
	R4	4	52.08%
Age	0–19	1	21.74%
	20–39	2	50.29%
	40–59	3	21.99%
	≥60	4	5.98%
Education level	Bachelor degree or above	1	4.98%
	Junior college	2	11.95%
	Senior high school	3	19.00%
	Middle school and below	4	49.49%
	Stage of compulsory education and before	-	14.58%
Occupation	Manufacturing	1	29.83%
	Service & business	2	19.02%
	Transportation	3	0.85%
	Agriculture & breeding	4	0.10%
	Construction	5	1.17%
	Unemployed	6	10.02%
	Other	-	19.87%
	Not yet of working age	-	19.14%

**Table 2:** The correlations between the supply–demand ratio of GHs/CHSCs and population characteristics.

Population Characteristics	Abbreviation	Supply–Demand Ratio of GHs	Supply–Demand Ratio of CHSCs
Proportion of population living in R4 houses	PR4	−0.273 **	0.387 **
Proportion of population younger than 40	PY40	−0.842 *	−2.349 **
Proportion of population above junior college	PJ	1.002 **	0.704 *
Proportion of population engaged in manufacturing industry	PM	−1.639 **	0.562 **
Proportion of population engaged in service and business industry	PB	2.276 **	0.603 *

Note: * indicates *p* < 0.05, ** indicates *p* < 0.01.

**Table 3:** The comparison between accessibility of GHs under actual and hypothetic locations.

Dimensions	Type	Class	Actual Accessibility of GHs	Accessibility of Hypothetic GHs
House type	R1	1	1.89	2.09
	R2	2	3.40	2.80
	R3	3	2.03	2.35
	R4	4	2.22	2.51
Age	0–19	1	2.56	2.60
	20–39	2	2.49	2.56
	40–59	3	2.68	2.59
	≥60	4	3.06	2.67
Education level	Bachelor degree or above	1	3.16	2.66
	Junior college	2	2.91	2.62
	Senior high school	3	2.89	2.65
	Middle school and below	4	2.32	2.53
Occupation	Manufacturing	1	1.81	2.35
	Service & business	2	3.21	2.81
	Transportation	3	2.09	2.25
	Agriculture & breeding	4	1.70	1.95
	Construction	5	2.33	2.49
	Unemployed	6	2.90	2.61

## References

[1] Wang H.H.X., Wang J.J. (2012). Effects of community-based general practitioners-led care for 12,864 patients with hypertension: Study of cardiovascular risk intervention—Hypertension (SCRI-HTN) in China. Eur. Heart J..

[2] Cutler D.M., Lleras-Muney A., Vogl T. (2011). Socioeconomic Status and Health: Dimensions and Mechanisms.

[3] Huerta M.U., Kallestal C. (2012). Geographical accessibility and spatial coverage modeling of the primary health care network in the Western Province of Rwanda. Int. J. Health Geogr..

[4] Owen K.K., Obregon E.J., Jacobsen K.H. (2010). A geographic analysis of access to health services in rural Guatemala. Int. Health.

[5] Zhang W., Cao K., Liu S., Huang B. (2016). A multi-objective optimization approach for health-care facility location-allocation problems in highly developed cities such as Hong Kong. Comput. Environ. Urban. Syst..

[6] Wang X., Yang H., Duan Z., Pan J. (2018). Spatial accessibility of primary health care in China: A case study in Sichuan Province. Soc. Sci. Med..

[7] Yin C., He Q., Liu Y., Chen W., Gao Y. (2018). Inequality of public health and its role in spatial accessibility to medical facilities in China. Appl. Geogr..

[8] Wu Y., Rowangould D., London J.K., Karner A. (2019). Modeling health equity in active transportation planning. Transp. Res. Part. D Transp. Environ..

[9] Chin M.H., King P.T., Jones R.G., Jones B., Ameratunga S.N., Muramatsu N., Derrett S. (2018). Lessons for achieving health equity comparing Aotearoa/New Zealand and the United States. Health Policy.

[10] Yichun Z., Jian F. (2017). Residential spatial differentiation of migrant population within the city: A case study of Shenzhen. Prog. Geogr..

[11] Lin L.Y., Zhu Y., Liang P.F., Xiao B.Y. (2014). The spatial patterns of housing conditions of the floating population in China based on the sixth census data. Geogr. Res.-Aust..

[12] Song Y., Zenou Y., Ding C. (2008). Let’s not throw the baby out with the bath water: The role of urban villages in housing rural migrants in China. Urban Stud..

[13] Feng C.C., Li T.J., Cao G.Z., Shen H.J. (2017). Housing outcomes of family migrants at the place of destination. Geogr. Res.-Aust..

[14] Tong D., Feng C.C., Deng J.J. (2011). Spatial evolution and cause analysis of urban villages: A case study of Shenzhen Special Economic Zone. Geogr. Res.-Aust..

[15] Shenzhen Statistics Bureau (2017). Shenzhen Statistical Yearbook 2017.

[16] Hao P., Sliuzas R., Geertman S. (2011). The development and redevelopment of urban villages in Shenzhen. Habitat Int..

[17] Guagliardo M.F. (2004). Spatial accessibility of primary care: Concepts, methods and challenges. Int. J. Health.

[18] Luo W., Qi Y. (2009). An enhanced two-step floating catchment area (E2SFCA) method for measuring spatial accessibility to primary care physicians. Health Place.

[19] Kanuganti S., Sarkar A.K., Singh A.P. (2016). Evaluation of access to health care in rural areas using enhanced two-step floating catchment area (E2SFCA) method. J. Transp. Geogr..

[20] McGrail M.R., Humphreys J.S. (2014). Measuring spatial accessibility to primary health care services: Utilising dynamic catchment sizes. Appl. Geogr..

[21] Langford M., Higgs G., Fry R. (2016). Multi-modal two-step floating catchment area analysis of primary health care accessibility. Health Place.

[22] Weibull J.W. (1976). An Axiomatic Approach to the Measurement of Accessibility. Reg. Sci. Urban. Econ..

[23] Joseph A.E., Bantock P.R. (1982). Measuring potential physical accessibility to general practitioners in rural areas: A method and case study. Soc. Sci. Med..

[24] Wang F. (2001). Explaining Intraurban Variations of Commuting by Job Proximity and Workers’ Characteristics. Environ. Plan. B Plan. Des..

[25] Wu C., Ye X., Du Q., Luo P. (2017). Spatial effects of accessibility to parks on housing prices in Shenzhen, China. Habitat Int..

[26] Gong J., Liu Y., Liu Y., Huang P., Li J. (2017). Evaluating the Evacuation and Rescue Capabilities of Urban Open Space from a Land Use Perspective: A Case Study in Wuhan, China. ISPRS Int. J. Geo-Inf..

[27] Luo W., Wang F. (2003). Measures of Spatial Accessibility to Health Care in a GIS Environment: Synthesis and a Case Study in the Chicago Region. Environ. Plan. B Plan. Des..

[28] McGrail M.R. (2012). Spatial accessibility of primary health care utilising the two steps floating catchment area method: An assessment of recent improvements. Int. J. Health Geogr..

[29] Kanuganti S., Sarkar A.K., Singh A.P. (2016). Quantifying Accessibility to Health Care Using Two-step Floating Catchment Area Method (2SFCA): A Case Study in Rajasthan. Transp. Res. Procedia.

[30] Li H., Liu Y. (2016). Neighborhood socioeconomic disadvantage and urban public green spaces availability: A localized modeling approach to inform land use policy. Land Use Policy.

[31] You H. (2016). Characterizing the inequalities in urban public green space provision in Shenzhen, China. Habitat Int..

[32] Luo J., Chen G., Li C., Xia B., Sun X., Chen S. (2018). Use of an E2SFCA Method to Measure and Analyse Spatial Accessibility to Medical Services for Elderly People in Wuhan, China. Int. J. Environ. Res. Public Health.

[33] Pan J., Liu H., Wang X., Xie H., Delamater P.L. (2015). Assessing the spatial accessibility of hospital care in Sichuan Province, China. Geospat. Health.

[34] Cheng G., Zeng X., Duan L., Lu X., Sun H., Jiang T., Li Y. (2016). Spatial difference analysis for accessibility to high level hospitals based on travel time in Shenzhen, China. Habitat Int..

[35] Zhu L., Zhong S., Tu W., Zheng J., He S., Bao J., Huang C. (2019). Assessing Spatial Accessibility to Medical Resources at the Community Level in Shenzhen, China. Int. J. Environ. Res. Public Health.

[36] Shah T.I., Bell S., Wilson K. (2016). Spatial Accessibility to Health Care Services: Identifying under-Serviced Neighbourhoods in Canadian Urban Areas. PLoS ONE.

[37] Tao Z., Cheng Y., Zheng Q., Li G. (2018). Measuring spatial accessibility to healthcare services with constraint of administrative boundary: A case study of Yanqing District, Beijing, China. Int. J. Equity Health.

[38] Gu X., Zhang L., Tao S., Xie B. (2019). Spatial Accessibility to Healthcare Services in Metropolitan Suburbs: The Case of Qingpu, Shanghai. Int. J. Environ. Res. Public Health.

[39] Tao L., Hui E.C.M., Wong F.K.W., Chen T. (2015). Housing choices of migrant workers in China: Beyond the Hukou perspective. Habitat Int..

